# Mitoxantrone is More Toxic than Doxorubicin in SH-SY5Y Human Cells: A ‘Chemobrain’ In Vitro Study

**DOI:** 10.3390/ph11020041

**Published:** 2018-05-05

**Authors:** Daniela Almeida, Rita Pinho, Verónica Correia, Jorge Soares, Maria de Lourdes Bastos, Félix Carvalho, João Paulo Capela, Vera Marisa Costa

**Affiliations:** 1 UCIBIO, REQUIMTE, Laboratory of Toxicology, Faculty of Pharmacy, University of Porto, Rua de Jorge Viterbo Ferreira, 228, 4050-313 Porto, Portugal; anadaniela_16@hotmail.com (D.A.); rita.pinho94@gmail.com (R.P.); vero_correi@hotmail.com (V.C.); jorge.emt.soares@gmail.com (J.S.); mlbastos@ff.up.pt (M.d.L.B.); felixdc@ff.up.pt (F.C.); joaoc@ufp.edu.pt (J.P.C.); 2 FP-ENAS (Unidade de Investigação UFP em Energia, Ambiente e Saúde), CEBIMED (Centro de Estudos em Biomedicina), Faculdade de Ciências da Saúde, Universidade Fernando Pessoa, 4249-004 Porto, Portugal

**Keywords:** mitoxantrone, doxorubicin, neurotoxicity, SH-SY5Y cells

## Abstract

The potential neurotoxic effects of anticancer drugs, like doxorubicin (DOX) and mitoxantrone (MTX; also used in multiple sclerosis), are presently important reasons for concern, following epidemiological data indicating that cancer survivors submitted to chemotherapy may suffer cognitive deficits. We evaluated the in vitro neurotoxicity of two commonly used chemotherapeutic drugs, DOX and MTX, and study their underlying mechanisms in the SH-SY5Y human neuronal cell model. Undifferentiated human SH-SY5Y cells were exposed to DOX or MTX (0.13, 0.2 and 0.5 μM) for 48 h and two cytotoxicity assays were performed, the 3-(4,5-dimethylthiazol-2-yl)-2,5-diphenyltetrazolium (MTT) reduction and the neutral red (NR) incorporation assays. Phase contrast microphotographs, Hoechst, and acridine orange/ethidium bromide stains were performed. Mitochondrial membrane potential was also assessed. Moreover, putative protective drugs, namely the antioxidants *N*-acetyl-l-cysteine (NAC; 1 mM) and 100 μM tiron, the inhibitor of caspase-3/7, Ac-DEVD-CHO (100 μM), and a protein synthesis inhibitor, cycloheximide (CHX; 10 nM), were tested to prevent DOX- or MTX-induced toxicity. The MTT reduction assay was also done in differentiated SH-SY5Y cells following exposure to 0.2 μM DOX or MTX. MTX was more toxic than DOX in both cytotoxicity assays and according to the morphological analyses. MTX also evoked a higher number of apoptotic nuclei than DOX. Both drugs, at the 0.13 μM concentration, caused mitochondrial membrane potential depolarization after a 48-h exposure. Regarding the putative neuroprotectors, 1 mM NAC was not able to prevent the cytotoxicity caused by either drug. Notwithstanding, 100 μM tiron was capable of partially reverting MTX-induced cytotoxicity in the NR uptake assay. One hundred μM Ac-DEVD-CHO and 10 nM cycloheximide (CHX) also partially prevented the toxicity induced by DOX in the NR uptake assay. MTX was more toxic than DOX in differentiated SH-SY5Y cells, while MTX had similar toxicity in differentiated and undifferentiated SH-SY5Y cells. In fact, MTX was the most neurotoxic drug tested and the mechanisms involved seem dissimilar among drugs. Thus, its toxicity mechanisms need to be further investigated as to determine the putative neurotoxicity for multiple sclerosis and cancer patients.

## 1. Introduction

Deaths after neoplasms have been increasing globally, rising from 7.58 million deaths in 2006 to 8.93 million deaths in 2016 [1]. Although new treatments have emerged recently, chemotherapy is still the most common treatment for most cancers [2,3]. Mitoxantrone (MTX) is an anticancer drug used in the treatment of metastatic breast cancer, non-Hodgkin’s lymphoma, and acute myeloid leukaemia in adults; while in combination regimens, it is indicated in the remission-induction treatment of blast crisis in chronic myeloid leukaemia, and in combination with corticosteroids for palliation (e.g., pain relief) related to advanced castrate-resistant prostate cancer [4]. Moreover, the U.S. Food and Drug Administration (FDA) approved its use in secondary progressive multiple sclerosis (SPMS), in progressive relapsing multiple sclerosis (MS), and for patients with worsening relapsing-remitting (RR) MS [5]. Various MTX dosage schedules can be used in cancer patients, although in adults with solid tumors, 12−14 mg/m^2^ every 3–4 weeks is usual [6]. MTX is given intravenously at a dose of 12 mg/m^2^ every 3 months to MS patients. Although a maximum cumulative lifetime dose of 140 mg/m^2^ should not be surpassed, recommendations exist of lower cumulative doses being given to MS patients as they seem more susceptible to cardiotoxicity [7]. Doxorubicin (DOX) is used in the treatment of the following cancers: metastatic breast cancer, advanced cancer of the ovary, Kaposi’s sarcoma and multiple myeloma [8]. DOX and MTX are both topoisomerase II inhibitors and exert their antineoplastic action by intercalating into DNA and producing both DNA strand-breaks and interstrand cross-links; they also interfere with RNA synthesis [4,8]. New drugs are emerging for cancer treatments, but chemotherapy is still the most common and well-characterized option in several cancers [3,9].

Although life expectancy is largely increasing due to the chemotherapeutic regiments available, serious side effects emerge after chemotherapy. Mielossupression and cardiotoxicity of MTX (and also of its analog, DOX) are common concerns among cancer treated patients [2,10,11,12]. However, data have shown that brain tissue is also susceptible to the toxicity of chemotherapeutic agents, despite presumption of blood-brain barrier (BBB) protection [13]. “Chemobrain” is the term used to describe the cognitive decline associated with chemotherapy. Persistent changes in cognitive function, including memory loss, distractibility, and difficulty in performing multiple tasks, have been observed in breast cancer survivors after treatment with chemotherapeutic agents, including DOX [14]. Women with breast cancer and treated with four cycles of DOX and cyclophosphamide had significant decreases in visuospatial skill and total cognitive scores, following chemotherapy [15]. In a broader study by the same authors, cognitive impairment was found in 23% of women prior to chemotherapy. Thereafter, they received the combination of DOX and cyclophosphamide or followed by a taxane. Significant decreases in the cognitive domains of visuospatial skill, attention, delayed memory, and motor function were observed after receiving chemotherapy, but improvements followed 6 months after the completion of chemotherapy [16]. The authors stated that having a breast cancer diagnosis can result in cognitive impairment and that chemotherapy may have a negative acute impact on cognitive function [16]. However, other data support long-term neurotoxic effects of chemotherapy. Long-term survivors of breast cancer or lymphoma, who had been treated with systemic chemotherapy (DOX being present in several of the systemic regimens taken), scored significantly lower on several neuropsychological tests compared to those treated with local therapy only, particularly in the domains of verbal memory, and psychomotor functioning; also they were in the lower quartile on the Neuropsychological Performance Index, and self-reported greater problems with working memory on the Squire Memory Self-Rating Questionnaire [17]. Some in vitro data demonstrate that both apoptosis and oxidative stress can be involved in DOX-induced neurotoxicity [18,19]. Moreover, treatment with DOX increases circulating level of tumor necrosis factor-alpha and leads to decline in mitochondrial respiration and mitochondrial protein nitration, being nitric oxide an important mediator of those effects in mice [20]. Regarding MTX, little data is available about its putative neurotoxicity, although some questions were placed in the past whether increased incidence of central nervous system (CNS) hemorrhages in patients with secondary acute promyelocytic leukemia were a consequence of MTX treatment [21]. As far as local neurological side effects are concerned, the administration of MTX caused very short-lived partial Jacksonian motor seizures; momentary light headache lasting 1–2 h and slight drowsiness lasting no more than 6 h in patients with recurrent glioblastomas enrolled for second tumor debulking with local positioning of a reservoir containing MTX [22]. In two patients, a hemorrhage occurred in the absence of any clinical deficits and vanished in both cases within a month [22]. Most importantly, the new use of MTX on MS may pose as an added risk for MTX-induced neurotoxicity, as dysregulation of the BBB is an early cerebrovascular abnormality seen in the MS patient brain [23], circumventing the important function of BBB as the retaining wall preventing drug passage into the CNS. In a work by Fulda et al. DOX and MTX were compared in neuroblastoma SK-*N*-SH cells and DOX was more toxic than MTX, according to monolayer proliferation assay [24]; however more studies are required. Therefore, our work aimed to determine the neurotoxicity profile of MTX and compare it with a known neurotoxic chemotherapeutic drug, DOX. SH-SY5Y cells are a frequent neuronal model used in neurotoxicity studies and are a subline of the parental line SK-*N*-SH. Those human cells were used herein as an in vitro neuronal model and several determinations were performed. Moreover, pharmacological active drugs were used as putative protectors, according to the data previously published on the putative DOX neurotoxic mechanisms [18,19,25,26,27].

## 2. Results

### 2.1. The Cytotoxicity of Mitoxantrone Was Significantly Higher Than That of Doxorubicin

**Figure 1:**
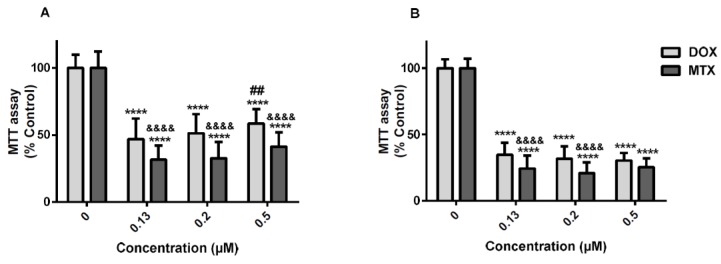
MTT reduction assay after exposure to 0.5, 0.2 and 0.13 μM DOX (light grey) or 0.5, 0.2 and 0.13 μM MTX (dark grey) after 24 h (**A**) or 48 h (**B**) in undifferentiated SH-SY5Y cells. Sterile PBS was used as control. Results are presented as mean ± SD of 23–35 wells, of 5–6 independent experiments. Statistical analyses were performed using two-way ANOVA followed by the Bonferroni *post-hoc* test(**** *p* < 0.0001 versus control; ^##^ *p* < 0.01 versus the same drug at 0.13 μM; ^&&&&^ *p* < 0.0001 MTX versus the same DOX concentration).

At 24 h, in the 3-(4,5-dimethylthiazol-2-yl)-2,5-diphenyl tetrazolium bromide (MTT) reduction assay, all concentrations of either DOX or MTX caused significant cytotoxicity when compared to control (Figure 1A), MTX being more cytotoxic than DOX [31.5 ± 10.5% (MTX) versus 46.9 ± 15.4% (DOX) for the lowest concentration; 32.6 ± 12.2% (MTX) versus 51.2 ± 14.3% (DOX) for the 0.2 μM intermediate concentration; and 41.4 ± 10.5% (MTX) versus 58.5 ± 10.8% (DOX) for the highest concentration (0.5 μM)]. Of note that, in the MTT assay, we could not find a concentration-dependent toxicity for MTX at both time-points tested. Moreover, DOX 0.5 μM was less toxic than the lowest DOX concentration (0.13 μM) tested at 24 h.

**Figure 2:**
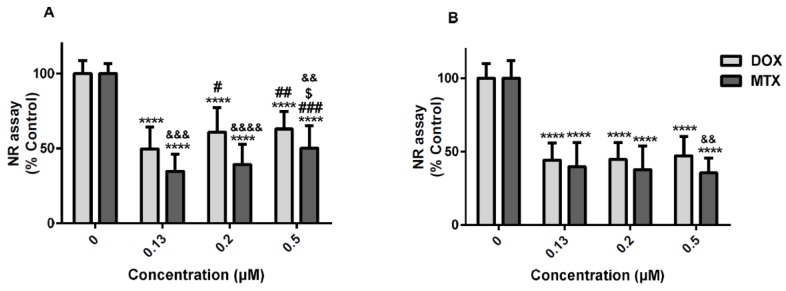
NR uptake assay after exposure to 0.5, 0.2 and 0.13 μM DOX (light grey) or 0.5, 0.2 and 0.13 μM MTX (dark grey) after 24 h (**A**) or 48 h (**B**) in undifferentiated SH-SY5Y cells. Sterile PBS was used as control. Results are presented as mean ± SD of 24–37 wells, of 5–7 independent experiments. Statistical analyses were performed using two-way ANOVA followed by the Bonferroni *post-hoc* test (^****^ *p* < 0.0001 versus control; ^#^ *p*<0.05 versus the same drug at 0.13 μM; ^##^ *p* < 0.01 versus the same drug at 0.13 μM; ^###^ *p* < 0.001 versus the same drug at 0.13 μM; ^&&^ *p* < 0.01 MTX versus 0.5 μM DOX; ^&&&^ *p* < 0.001 MTX versus 0.13 μM DOX; ^&&&&^ *p* < 0.0001 MTX versus 0.2 μM DOX; ^$^ *p* < 0.05 versus same molecule at concentration 0.2 μM).

At the 48 h time-point, MTX caused the highest toxicity at concentrations of 0.13 μM and 0.2 μM, when compared to DOX in the same concentrations (Figure 1B). At 24 h, significant differences were observed between the two molecules, in the neutral red (NR) uptake assay, MTX being more cytotoxic than DOX (Figure 2A). At 48 h, significant differences between DOX and MTX were only found at 0.5 μM (DOX: 47.2 ± 13.3%; MTX: 35.6 ± 10.1%) (Figure 2B). Additionally, in the NR uptake assay and following a 24-h exposure, the lower concentration (0.13 μM) of both DOX and MTX was more toxic than the highest concentration tested (0.5 μM) (Figure 2A). Meanwhile, this difference was not verified at 48 h (Figure 2B).

### 2.2. Mitoxantrone Led to Cellular Damage in SH-SY5Y Cells, with Signs of Apoptosis Most Evident at the Lowest Concentration after a 48-h Exposure

**Figure 3:**
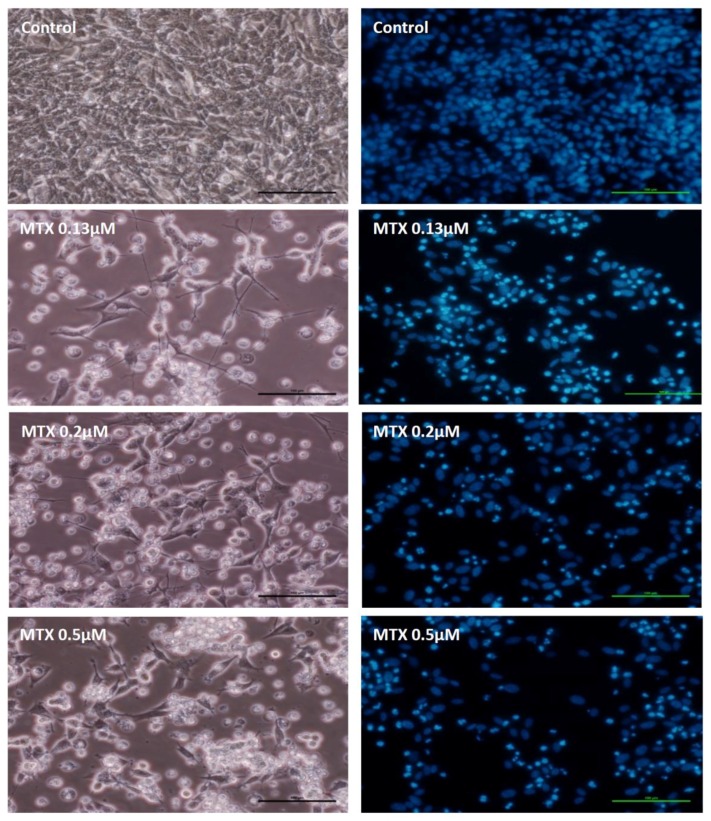
Phase-contrast microphotographs (left column) of undifferentiated SH-SY5Y cells exposed to PBS (control) or 0.13 μM MTX, 0.2 μM MTX and 0.5 μM MTX. Right side, fluorescence microscopy (Hoechst 33258 staining) of undifferentiated SH-SY5Y cells incubated with PBS (control) or 0.13 μM, 0.2 μM and 0.5 μM MTX. The microphotographs were taken after a 48-h exposure to the various conditions. Images are representative of two independent experiments with at least two wells (scale bar represents 100 μm).

A decrease in cell density was observed in all MTX-treated cells with a typical loss of shape and loss of neurites, at 48 h (Figure 3). The neurotoxic phenomenon was more expressive than the one observed in cells incubated with MTX for 24 h (data not shown). Cell number was substantially decreased after MTX treatment, as seen in the Hoechst staining (Table 1). Additionally, the lower concentration of MTX (0.13 μM) had a higher number of cells with apoptotic nuclear morphology, namely nuclear fragmentation, as well as chromatin condensation than the other MTX concentrations tested (Figure 3 and Table 1).

**Figure 4:**
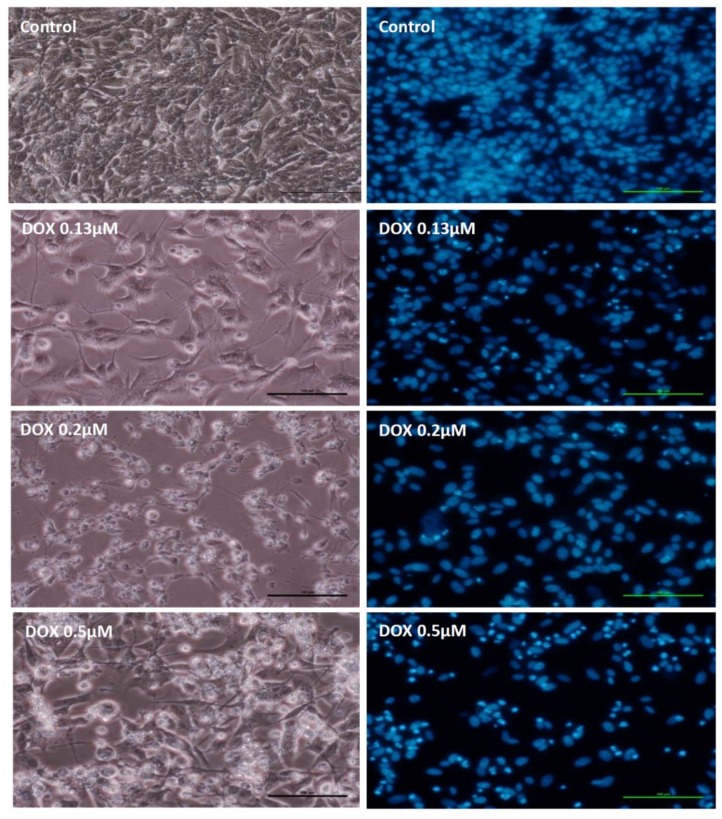
Phase-contrast microphotographs (left column) of undifferentiated SH-SY5Y cells incubated with PBS (control) or 0.13 μM, 0.2 μM and 0.5 μM DOX. Right side, fluorescence microscopy (Hoechst 33258 staining) of undifferentiated SH-SY5Y cells incubated with PBS (control) or 0.13 μM, 0.2 μM and 0.5 μM DOX. The microphotographs were taken after a 48-h exposure to the various conditions. Images are representative of two independent experiments with at least two wells (scale bar represents 100 μm).

The toxicity observed at 48 h (Figure 4) was higher after DOX exposure than at 24 h at the same concentrations (data not shown). At 48 h, DOX caused a substantial decrease in cell density when compared to control and many cells treated with DOX had rounded appearance without neuritis (Figure 4). In the fluorescence microscopy photographs, nuclear fragmentation and chromatin condensation were observed after a 48-h exposure to DOX, with a higher number of apoptotic cells at the highest concentration tested (0.5 μM) (Figure 4 and Table 1).

### 2.3. Mitoxantrone and Doxorubicin Caused Apoptosis in Undifferentiated SH-SY5Y Cells

**Figure 5:**
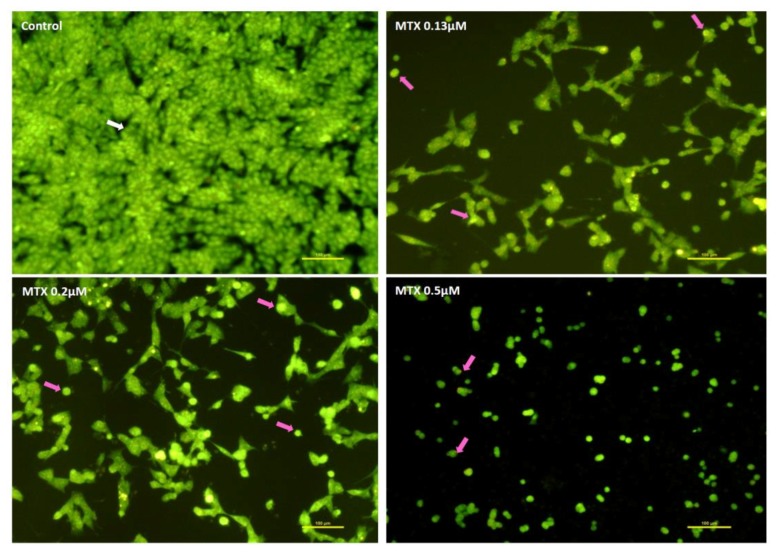
Representative fluorescence microscopic photos following acridine orange/ethidium bromide staining in undifferentiated SH-SY5Y cells after a 48-h incubation with MTX (0.13 μM; 0.2 μM; 0.5 μM) and control cells. White arrow: viable cells; pink arrows: cells with signs of apoptosis. Images are representative of two independent experiments with at least two wells. Scale bar represents 100 μm.

**Figure 6:**
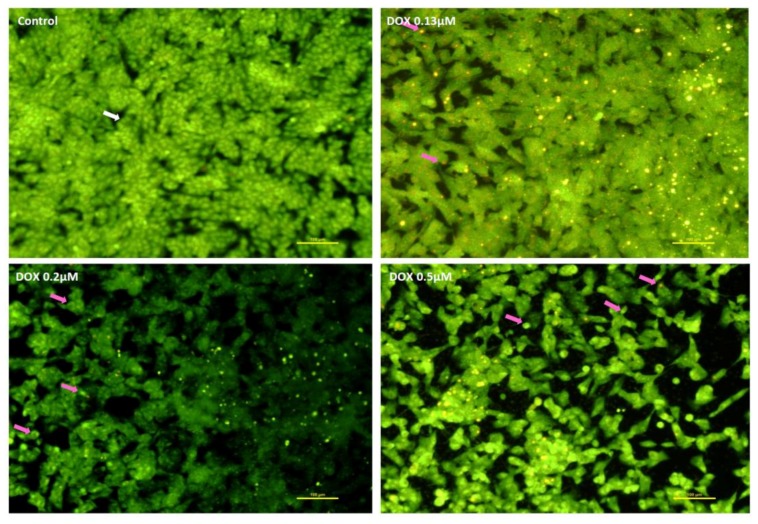
Representative fluorescence microscopic photos following acridine orange/ethidium bromide staining in undifferentiated SH-SY5Y cells after a 48-h incubation with DOX (0.13 μM; 0.2 μM; 0.5 μM) and control cells. White arrow: viable cells; pink arrows: cells with signs of apoptosis. Images are representative of two independent experiments with at least two wells. Scale bar represents 100 μm.

In Figure 5 and Figure 6, cells with well-colored green and large nuclei (white arrow) were seen in the control living cells. In SH-SY5Y cells exposed to MTX for 48 h, cells with condensed green nucleus (pink arrows), indicative of early apoptotic cells, were observed with very few adherent cells in the field (Figure 5). After exposure to 0.5 μM MTX, the lack of neurites was evident, when compared to the two lower concentrations (Figure 5). In Figure 6, cells incubated with DOX for 48 h are shown, and several red dots of nuclear condensation within the cells or condensed nuclei, both signs of apoptosis (pink arrows) were noteworthy in all concentrations. At 48 h, a higher number of cells per field was observed in DOX-exposed cells when compared with MTX-incubated SH-SY5Y cells, in the same concentrations.

### 2.4. Both Doxorubicin and Mitoxantrone Caused a Decrease in the Mitochondria Potential of Neuronal Cells at 0.13 μM

**Figure 7:**
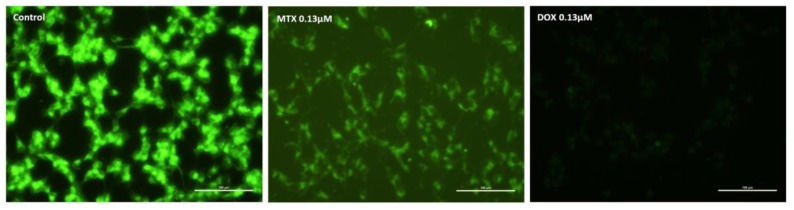
Microphotographs showing the mitochondrial transmembrane potential of undifferentiated SH-SY5Y control cells, and cells incubated with 0.13 μM MTX or 0.13 μM DOX for 48 h. Images are representative of two independent experiments with at least two wells. Scale bar represents 100 μm.

The transmembrane mitochondrial potential of undifferentiated SH-SY5Y cells following a 48-h exposure can be seen in Figure 7. Control cells were bright green. The lower concentration of MTX (0.13 μM) led to a total depolarization of the mitochondria, when compared to the control cells. Meanwhile, 0.13 μM DOX also caused evident depolarization of cells after a 48-h exposure. 

### 2.5. Tiron, an Antioxidant, was the Only Drug That Partially Prevented the Cytotoxicity of Mitoxantrone in the Neutral Red Uptake Assay

**Figure 8:**
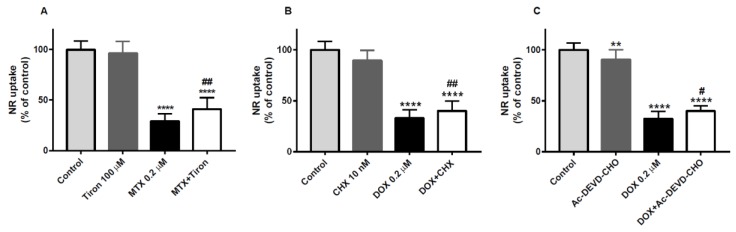
(**A**) NR uptake assay in undifferentiated SH-SY5Y cells proving the protective effect of tiron against MTX neurotoxicity. Four conditions were tested: control (with PBS); 100 μM tiron; 0.2 μM MTX; 0.2 μM MTX + 100 μM tiron. Results are presented as mean ± SD of 17–24 wells of 3–4 independent experiments. Statistical analyses were performed using the one-way ANOVA, followed by the Tukey’s *post hoc* test (**** *p* < 0.0001 versus control; ^##^ *p* < 0.01 versus 0.2 μM MTX). (**B**) NR uptake assay in undifferentiated SH-SY5Y cells showing the protective effect of cycloheximide (CHX) against DOX neurotoxicity. Four conditions were tested: control (with PBS); 10 nM CHX; 0.2 μM DOX; 0.2 μM DOX + 10 nM CHX. Results are presented as mean ± SD of 14–18 wells of 3 independent experiments. Statistical analyses were performed using the Kruskal–Wallis test, followed by the Dunn’s *post hoc* test (**** *p* < 0.0001 versus control; ^##^ *p* < 0.01 versus 0.2 μM DOX). (**C**) NR uptake assay in undifferentiated SH-SY5Y cells proving the protective effect of Ac-DEVD-CHO against DOX neurotoxicity. Four conditions were tested: control (with PBS); 100 μM Ac-DEVD-CHO; 0.2 μM DOX; 0.2 μM DOX + 100 μM Ac-DEVD-CHO. Results are presented as mean ± SD of 15–18 wells of 3 independent experiments. Statistical analyses were performed using the one-way ANOVA, followed by the Tukey’s *post hoc* test (** *p* < 0.01, **** *p* < 0.0001 versus control; ^#^ *p* < 0.05 versus 0.2 μM DOX).

Tiron was able to partially prevent the MTX-induced cytotoxicity in undifferentiated SH-SY5Y cells in the NR uptake assay, but not in the MTT reduction assay at 48 h (data not shown). In the MTT reduction assay, the values obtained after exposure to 0.2 μM MTX, either alone or in combination with 100 μM tiron, were always lower than control and not different between themselves (data not shown). Moreover, 100 μM tiron alone did not cause any cellular cytotoxicity. The MTX 0.2 μM exposed cells (29.0 ± 7.6%) had lower levels of NR uptake than the control cells (100.0 ± 8.4%), which revealed its neurotoxic nature (Figure 8A). However, when compared with MTX + tiron (41.1 ± 11.3%), 100 μM tiron revealed to be protective (Figure 8A). Moreover, there was no significant difference between the 100 μM tiron condition (96.2 ± 11.6%) and control cells (100.0 ± 8.4%) (Figure 8A). *N*-acetyl-l-cysteine (NAC, 1 mM), also an antioxidant, was not able to avoid the cytotoxicity induced by 0.2 μM MTX at 48 h in undifferentiated SH-SY5Y cells, neither in the MTT reduction assay nor in the NR uptake assay (data not shown). Also, neither 10 nM CHX nor 100 μM Ac-DEVD-CHO, an inhibitor of caspase-3/7, were able to prevent the cytotoxicity observed after exposure to 0.2 μM MTX, in the MTT reduction assay or in the NR uptake assay (data not shown).

### 2.6. Cycloheximide, a Protein Synthesis Inhibitor, and Ac-DEVD-CHO, an Inhibitor of Caspase-3, Partially Counteracted the Doxorubicin-Induced Toxicity 

CHX was able to partially prevent DOX-induced cytotoxicity in undifferentiated SH-SY5Y cells in the NR uptake assay (Figure 8B), but not in the MTT assay at 48 h (data not shown). In the NR uptake assay, the values after exposure to 0.2 μM DOX (32.9 ± 8.4%) were lower than the control (100.0 ± 8.2%, Figure 8B). The DOX + CHX (40.2 ± 9.5%) condition had also lower values than those of control, but significantly higher than that obtained after exposure to 0.2 μM DOX alone, revealing the neuroprotective action of CHX. In the NR uptake assay, 10 nM CHX exposed cells (93.8 ± 8.1%) did not differ to the respective control (Figure 8B: 100.0 ± 8.2%).

The caspase-3 inhibitor was able to provide partial neuroprotection against 0.2 μM DOX toxicity in undifferentiated SH-SY5Y cells in the NR uptake assay (Figure 8C). DOX + Ac-DEVD-CHO had significantly higher NR uptake values (40.1 ± 5.0%) than those of 0.2 μM DOX alone (32.3 ± 7.2%), demonstrating that the inhibitor had a partial neuroprotective action against the anticancer drug toxicity. Of note, 100 μM Ac-DEVD-CHO (89.3 ± 10.8%) caused some toxicity *per se* when compared to control cells (100.0 ± 6.7%). In the MTT reduction assay, 0.2 μM DOX and DOX + Ac-DEVD-CHO conditions had similar values (data not shown).

One mM NAC was not able to counteract the cytotoxicity induced by 0.2 μM DOX at 48 h in undifferentiated SH-SY5Y cells, neither in the MTT reduction assay nor in the NR uptake assay (data not shown). Moreover, tiron was not protective against DOX-induced neurotoxicity, in both cytotoxicity assays performed, as the 0.2 μM DOX and DOX + tiron conditions did not show significant differences among themselves (data not shown). 

### 2.7. Doxorubicin Caused Greater Cytotoxicity in Undifferentiated SH-SY5Y Cells than in Differentiated Cells According to the MTT Reduction Assay

**Figure 9:**
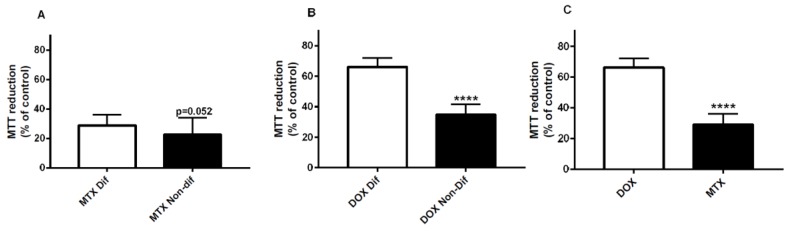
MTT reduction assay after exposure to 0.2 μM MTX (**A**) or 0.2 μM DOX (**B**) in differentiated or undifferentiated SH-SY5Y cells. Four conditions were tested: 0.2 μM MTX in differentiated cells, 0.2 μM MTX in undifferentiated cells, 0.2 μM DOX in differentiated cells and 0.2 μM DOX in undifferentiated cells. Values are expressed as percentage of control and are presented as mean ± SD. The results were obtained from 12–24 wells, from 2–4 independent experiments. Data were statistically analyzed using the unpaired *t*-test (**** *p* < 0.0001 versus differentiated cells treated with drug 0.2 μM). (**C**) MTT reduction assay after exposure to MTX or DOX in differentiated SH-SY5Y cells. Two conditions were tested: 0.2 μM DOX and 0.2 μM MTX. Values are expressed as percentage of control and are presented as mean ± SD. The results were obtained from 12 wells and 2 independent experiments. Data were statistically analyzed using the unpaired *t*-test (**** *p* < 0.0001 versus 0.2 μM DOX).

Following a 48-h exposure, differentiated SH-SY5Y cells exposed to 0.2 μM MTX presented slightly higher values in the MTT reduction test than undifferentiated cells, but with no statistical significance (differentiated cells: 29.0 ± 7.1%; undifferentiated cells: 22.6 ± 11.5%) (Figure 9A). At 48 h, the differentiated cells exposed to 0.2 μM DOX presented substantially higher MTT reduction values (66.2 ± 6.0%) than in undifferentiated cells at the same conditions (34.8 ± 6.8%) (Figure 9B).

### 2.8. Mitoxantrone is More Neurotoxic than Doxorubicin in Differentiated SH-SY5Y Cells in the MTT Reduction Assay

Both DOX and MTX caused neurotoxicity in differentiated SH-SY5Y cells after a 48-h exposure (Figure 9C). In the MTT reduction assay, 0.2 μM MTX (29.0 ± 7.1%) was more cytotoxic than 0.2 μM DOX (66.2 ± 6.0%) in differentiated SH-SY5Y cells (Figure 9C).

## 3. Discussion

This study revealed the following major findings: (1) MTX and DOX caused a time-dependent cytotoxicity in the NR uptake and in the MTT reduction assays, in undifferentiated SH-SY5Y cells; (2) MTX was shown to cause greater cytotoxicity at 24 h than DOX in undifferentiated SH-SY5Y cells; (3) MTX caused greater morphological damage, with lower cell density and neurites loss, when compared to DOX; (4) both drugs caused signs of apoptosis, in particular MTX, as both revealed by Hoechst and the ethidium bromide and acridine orange stains; (5) the lower concentration of DOX and MTX (0.13 μM) caused significant mitochondrial depolarization in undifferentiated SH-SY5Y cells; (6) tiron, an antioxidant, partially avoided the neurotoxicity exerted by MTX on undifferentiated SH-SY5Y cells in the NR uptake assay; (7) CHX and the caspase inhibitor, Ac-DEVD-CHO, were partially neuroprotective against the cytotoxicity caused by DOX on undifferentiated SH-SY5Y cells in the NR uptake assay; (8) in the MTT reduction assay, the cytotoxicity caused by MTX was similar, regardless of SH-SY5Y cells differentiation status, whereas DOX was more toxic in undifferentiated SH-SY5Y cells; and (9) in differentiated SH-SY5Y cells, MTX was shown to be more neurotoxic than DOX, according to the MTT reduction assay.

Drugs used in chemotherapy, such as DOX and MTX, may be responsible for neuronal damage with consequent neurotoxicity [22,28]. Persistent changes in cognitive function, including memory loss, distractibility, and difficulty in performing multiple tasks, have been observed in breast cancer survivors after chemotherapy, namely with DOX [15,16,17]. One could argue that BBB works as a protective barrier avoiding the entrance of several compounds and it is generally accepted that efflux transporters, namely P-glycoprotein and breast cancer resistance protein (BCRP) present in BBB, would extensively prevent the entrance of DOX and MTX to the CNS [29,30]. However, pharmacokinetic data obtained *post mortem* of treated patients show that both drugs are present in the brain [31,32]. Moreover, although MTX and DOX are given in very different doses to cancer patients, those doses are considered equivalent in the clinical practice [33]. In plasma of cancer-treated patients, DOX ranged between 0.04 to 1.16 μM and MTX levels were between 0.04 to 0.3 μM [34,35,36,37,38,39], while it is expected that the brain may be exposed to lower concentrations of those found in the plasma. We observed that both MTX and DOX, at clinically relevant concentrations, caused a high degree of cytotoxicity in undifferentiated SH-SY5Y cells in both the MTT reduction and NR uptake assays, MTX being more cytotoxic. The greater toxicity of MTX herein may be due to its superior lipophilicity [40], making this drug more easily permeable and accumulated inside the cells. A study published in 2005 also reported that MTX is also more cytotoxic than DOX in two immortalized cell lines (NIH 3T3 and B14), using the MTT reduction assay [41]. However, other authors reported that, in cardiac (H9c2) and breast cancer (MTLn3) cells, DOX was slightly more cytotoxic than MTX, according to the trypan blue exclusion technique [42]. Nevertheless, the trypan blue exclusion technique may present some subjectivity regarding the cell-counting operator and it does not allow counting the cells that completely disintegrated. Still, the toxicity of these two drugs seems to be dependent on the cellular model. Lopes et al. found in primary rat cortical neurons that DOX (0.1 and 0.5 μM) caused a substantial degree of toxicity (higher than toxic dose 50) when tested for 48 h according to the MTT reduction assay [19]. Meanwhile, in the same study, 10 μM DOX was less toxic than the lower concentrations tested (0.1 or 0.5 μM). That is in agreement with our results in undifferentiated SH-SY5Y cells, where 0.13 μM of DOX was more toxic than 0.5 μM both in the MTT and NR assays at 24 h. We used two cytotoxicity assays, the NR uptake and the MTT reduction assays, and several morphological evaluations or stains. Most authors agree that using several cytotoxicity tests, with different inherent mechanisms can help elucidate the underlying cytotoxicity of the drugs tested [19,43,44]. Nonetheless, herein there were no major differences between the two cytotoxicity tests performed in each drug; however, MTX is more toxic in both time points and concentrations and morphological evaluation corroborates the highest MTX cytotoxicity.

DOX and MTX kill cancer cells by intercalating their DNA and inhibiting topoisomerase II [4]. However, in non-target sites, such as the brain, these drugs also cause damage [14,15]. As neurons are post-mitotic cells, their injury can cause irreversible loss [14]. Regarding the mechanisms involved in the putative neurotoxicity of DOX or MTX, data are still scarce. In this work, undifferentiated SH-SY5Y cells were sensitive to DOX and MTX in a time-dependent manner and signs of apoptosis were confirmed by two stains. In Hoechst staining, MTX and DOX in all the conditions tested, caused signs of apoptosis, although more evident in 0.5 μM DOX and in 0.13 μM MTX, at 48 h. The acridine orange/ethidium bromide staining confirmed that both drugs caused apoptosis in the concentrations tested at 48 h. In primary cortical neurons, DOX caused signs of apoptosis, albeit dependent on DOX concentration. In those cells, only the lowest concentrations tested (0.1 and 0.5 μM) showed activation of caspase 3 and DNA fragmentation [19]. Loss of cell adhesion, loss of nuclear envelope, nuclear fragmentation and decrease in cell size are typical signs of apoptosis [45] and we observed those effects in the DOX and MTX-exposed undifferentiated SH-SY5Y cells. In mice, the oxidative damage caused by DOX was capable of enhancing the expression of pro-apoptotic factors, such as BAD protein, and decreasing the expression of Bcl-2 anti-apoptotic protein families, which would consequently lead to mitochondrial membrane loss of potential, promote the release of cytochrome *c* and apoptosis [46]. In the present study, the effects on mitochondrial membrane potential were evaluated at the lowest concentration (0.13 μM) and evident mitochondrial depolarization was observed, which agrees with the ability of both drugs to trigger mitochondrial damage. Rats treated with seven weekly injections of vehicle (subcutaneous, saline solution) or DOX (subcutaneous, 2 mg·kg^−1^), and then sacrificed one week after the last administration had brain mitochondrial fractions isolated, and the authors found that DOX treatment induced an increase in thiobarbituric acid-reactive substances and vitamin E levels and a decrease in reduced glutathione content and aconitase activity, while potentiated the mitochondrial permeability transition pore opening induced by calcium [47]. Also, DOX-induced caspase-8 and -3 activity increases and decrease in mitochondrial potential in several types of primary neuronal cells, although DOX impact was dependent on the cells’ development stage [26]. Those works demonstrated that DOX causes mitochondrial affection and oxidative stress. Lopes and colleagues also focused on neuronal oxidative stress caused by DOX, in primary cortical neurons, and found that it decreased glutathione and increased reactive species and quinoprotein levels [18]. The decrease of antioxidant defenses, such as glutathione, and increase of oxidative stress seem to contribute to neuronal damage [13], like it happens in the heart after DOX exposure [10]. Taking into account the ability of DOX to promote oxidative stress, we sought to prevent the neurotoxicity of DOX and MTX in undifferentiated SH-SY5Y cells using two antioxidants. NAC works as precursor in the synthesis of glutathione and acts as an antioxidant [48,49]; however, in this work it failed to provide any protection against 0.2 μM MTX or DOX toxicity. Rat cortical astrocytes were previously used to test the protective effect of NAC against DOX-induced toxicity [50]. NAC, when pre-incubated at a concentration of 5 mM, was able to reduce lipid peroxidation induced by DOX (10 mg/mL), and also partially counteracted DOX-induced cytotoxicity, when evaluated by the MTT reduction assay. The concentrations used, as well as the cellular model, may explain the differences observed in our work, where NAC showed no protective effect against DOX. Indeed, in rat cortical neurons, DOX only decreased glutathione cellular levels when cells were exposed to 0.5 μM for 24 h, not significantly altering this parameter at 0.1, 5 and 20 μM concentrations, possibly due to the overproduction of peroxynitrite seen in that concentration (0.5 μM) and not significantly seen in the others [18]. The putative protective action of KU-55933, an inhibitor of kinase ataxia-telangiectasia mutated (ATM), a protein engaged in DNA damage repair, was studied in SH-SY5Y cells exposed to DOX. KU-55933 inhibited the cell death induced by H_2_O_2_ [0.5 mM and 1 mM in undifferentiated and retinoic acid (RA)-differentiated SH-SY5Y cells, respectively] or DOX (0.25 and 1 μM in undifferentiated- and RA-differentiated SH-SY5Y cells, respectively) in undifferentiated and RA-differentiated SHSY5Y cells, with a more pronounced effect in the latter cell phenotype. Furthermore, this ATM inhibitor attenuated the DOX- but not H_2_O_2_-induced caspase-3 activity increase in both SH-SY5Y cells, showing that oxidative stress is not the unique mechanism of DOX-induced neurotoxicity [51]. Another antioxidant, tiron 100 μM, a superoxide anion radical scavenger, was tested here. Tiron was unable to prevent the toxicity of DOX, but it partially avoided the toxicity induced by MTX. Interestingly, oxidative stress appears to be involved in the cytotoxicity mechanism of MTX in our neuronal model; however, MTX, in other cellular models, namely H9c2 and MTLn3, did not led to reactive oxygen species increase, unlike DOX [42]. However, late formation of reactive species is observed after MTX incubation following mitochondrial affectation [52]. Our results advocate for MTX-induced toxicity to mitochondria in this model. Superoxide anion radical is mainly produced in mitochondria and MTX interferes in both the ATP levels and in ATP synthase expression and activity, leading to late reactive species formation [52,53]. Regarding DOX, other reactive oxygen or nitrogen species (not the superoxide anion radical), may be involved in the putative oxidative stress promoted by DOX. Truthfully, the activation of nitric oxide synthase has been reported in cortical neurons incubated with DOX, thus contributing to the formation of nitric oxide and subsequently reactive nitrogen species [18].

Herein, DOX and MTX caused apoptotic nuclei in undifferentiated SH-SY5Y cells. These results are in accordance to previous studies showing that MTX causes an increase in apoptotic cells at concentrations as low as 0.3 ng/mL in postmitotic sympathetic neurons after a 24-h exposure [54] and that DOX causes necrosis and apoptosis in rat cortical neurons at concentrations in the μM range [19]. We demonstrated that 10 nM CHX, a protein synthesis inhibitor, partially avoided the toxicity caused by 0.2 μM DOX, in line to a previous finding showing that CHX was able to totally revert the cell death caused by 0.5 μM DOX in cortical rat neurons [19]. Based on these data, even in different cellular models, it is possible to conclude that DOX-induced cytotoxicity is dependent on de novo protein synthesis. On the other hand, CHX was not able to lessen MTX-induced cytotoxicity in our neuronal model, indicating different toxicity mechanisms for DOX and MTX. 

Programmed cell death may be dependent or independent of caspases. A caspase-3 inhibitor, Ac-DEVD-CHO, partially reverted the neurotoxicity of 0.2 μM DOX in undifferentiated SH-SY5Y cells. In rat cortical neurons, exposure for 24 h to 0.1 and 0.5 μM DOX was found to increase caspase-3 activity [19], and the caspase-3 inhibitor, Z-DEVD-fmk, inhibited this effect, although it did not prevent cell death [19]. In the case of MTX, the caspase-3 inhibitor had no protective effect on undifferentiated SH-5YSY cells, although MTX was shown to activate caspase-3 in H9c2 cells [52]. However, since MTX is more lipophilic than DOX [41] and led to a higher number of apoptotic nuclei in undifferentiated SH-SY5Y cells in our work, the concentration of the caspase inhibitor may have been insufficient to prevent the toxicity of MTX or a non-caspase mediated apoptosis may also have been triggered. Actually, in two immortalized cell lines (NIH 3T3 and B14 cells) both DOX and MTX activated caspase-3 and the inhibitor Ac-DEVD-CHO did not show any significant effect on drug cytotoxicity either [41]. Moreover, DOX has been shown to activate the apoptosis inducing factor (AIF), which leads to caspase-3 independent apoptosis [25].

To assess whether there would be significant differences in the cytotoxicity promoted by DOX and MTX in undifferentiated *versus* differentiated SH-SY5Y cells, the cytotoxicity of both 0.2 μM DOX and 0.2 μM MTX was assessed at 48 h using the MTT reduction test. The MTT reduction test has been the most described cytotoxicity assay in the literature and in our results with undifferentiated SH-SY5Y cells, it was the most sensitive assay towards both DOX and MTX neurotoxicity. In our work, DOX was more neurotoxic in undifferentiated SH-SY5Y cells than in differentiated cells, revealing that the cellular modifications following differentiation may be protective to differentiated SH-SY5Y cells. These results are in line with those published earlier by Jantas and coworkers, who reported that undifferentiated SH-SY5Y cells were more sensitive to DOX (concentration range 0.1 to 5 μM) cytotoxicity in the lactate dehydrogenase release assay, and that DOX increased caspase 3 activity in undifferentiated, but not in RA-differentiated SH-SY5Y cells (cells differentiated for 7 days with 10 μM RA) [27]. Accordingly, we saw that the caspase-3 inhibitor, Ac-DEVD-CHO, could attenuate DOX toxicity in undifferentiated SH-SY5Y cells. The same group published another report revealing that DOX-evoked cell death in the MTT test (cells exposed 24 h to 0.25 or 1 μM) was attenuated by specific activators of group III metabotropic glutamate receptors in undifferentiated, but not in RA-differentiated SH-SY5Y cells [25]. In fact, cells subjected to the differentiation protocol undergo several biochemical changes. In particular, our group has shown that SH-SY5Y cells gain dopaminergic characteristics and suffer a strong slowdown in cell division capacity [48,55]. At the biochemical level, an increase in the density of dopamine receptors D2 and D3 on the cell surface of differentiated cells, an increase in tyrosine hydroxylase expression and in the dopamine transporter, rendering them dopaminergic neuronal characteristics [48,55]. RA differentiated SH-SY5Y cells were shown to be more resistant to apoptosis via increasing the expression of Bcl-2 anti-apoptotic protein family (cells differentiated for 4 days with 10 μM RA) [56]. In another neuroblastoma cell line, SK-*N*-SH, cells differentiated with RA (3 μM) or 4b-phorbol 12-myristate 13-acetate (PMA, 20 nM), a compound chemically related to 12-O-tetradecanoylphorbol 13-acetate (TPA), were more resistant to apoptosis than undifferentiated cells. PMA treated cells had an increased expression of Bcl-2 and RA treatment increased Bcl-x_L_, and these increases of anti-apoptotic proteins show how differentiation can render cells more resistant to apoptotic stimuli [57], namely those possibly caused by DOX herein. Additionally, RA differentiation induces a dramatic increase in the energy metabolism of SH-SY5Y cells, and shifts the dependence on energy production from glycolysis to oxidative phosphorylation [58,59] advocating that DOX causes energetic stress, and differentiated SH-SY5Y are more resilient cells possibly because they rely more on mitochondrial energy production. Actually, in murine cardiac HL-1 cells, ATP levels and glycolytic fluxes were significantly reduced after DOX treatment [60]. When comparing to MTX cytotoxicity in both undifferentiated and differentiated cells, no significant differences were seen but a tendency occurred towards a higher toxicity in undifferentiated cells. Neurons are very sensitive to mitochondrial toxins [61] and MTX is a mitochondrial toxin [43,52,53], even in mainly glycolytic cells [52]. This extensive neurotoxicity combined with its higher lipophilicity, make MTX a more dangerous drug to the brain than DOX. These data combined with its new use in MS, broadens MTX neurotoxic potential since BBB in MS patients is largely affected [23]. Thus, potential deleterious effects of MTX in the brain should not be overlooked or regarded as a natural path in the disabling and incurable MS and MTX neurotoxic effects should be further studied.

## 4. Materials and Methods

### 4.1. Materials

MTX, DOX, trypsin-EDTA solution, trypan blue solution 0.4% (*w*/*v*) and Dulbecco’s modified Eagle medium (DMEM) high glucose, sodium bicarbonate, 3-(4,5-dimethylthiazol-2-yl)-2,5-diphenyl tetrazolium bromide (MTT), neutral red (NR) solution, Hoechst 33258 solution, 3,3′-dihexyloxacarbocyanine iodide (DiO6), dimethyl sulfoxide (DMSO), RA, TPA, NAC, tiron, CHX, Ac-DEVD-CHO, an inhibitor of caspase-3/7, and paraformaldehyde were obtained from Sigma-Aldrich (Taufkirchen, Germany). Human neuroblastoma SH-SY5Y cells were obtained from the European Collection of Cell Cultures (Sigma-Aldrich, Taufkirchen, Germany). All sterile plastic material was obtained from Corning Costar (Corning, NY, USA). Penicillin/streptomycin (10.000 units/mL/10.000 μg/mL) and the phosphate buffer solution (PBS) without calcium and magnesium were obtained from Biochrom (Berlin, Germany). Fetal bovine serum (FBS), PBS with calcium and magnesium and Hank’s balanced salt solution (HBSS) were obtained from Gibco (Paisley, UK).

### 4.2. Cell Culture 

Human SH-SY5Y neuroblastoma cells are a commonly used neuronal model for the study of neurotoxicity, as they maintain several neuron markers [55]. SH-SY5Y cells were grown in complete DMEM that consisted of DMEM supplemented with 10% (*v*/*v*) FBS and 1% (*v*/*v*) of penicillin/streptomycin. Cells were cultured and maintained at 37 °C in a 5% CO_2_ incubator (Heraeus, Hanau, Germany) throughout all procedures. Stock cultures of SH-SY5Y cells were maintained in 25 cm^3^ flasks and grown until confluence (80–90% confluence). Cells were washed with PBS, trypsinized (trypsin/EDTA) and counted following trypan blue staining using a Fuchs-Rosenthal counting chamber. The cell suspension was then seeded in multi-well plates at a density of 50,000 cells/cm^2^. All experiments were done using cells from passage 25 to 40.

### 4.3. Undifferentiated SH-SY5Y Cells

After seeding the cells in plates, they were maintained for 24 h to allow them to attach, and then exposed to DOX or MTX (0.13; 0.2; 0.5 μM) for 24 or 48 h. NR uptake and MTT reduction assays, phase contrast microscopy, Hoechst stain, ethidium bromide and acridine orange stain, mitochondrial membrane potential evaluation were subsequently done.

For testing putative protectors against the toxicity of MTX or DOX, undifferentiated SH-SY5Y cells were pre-incubated with 1 mM NAC, 100 μM tiron, 10 nM CHX, or 100 μM of the caspase 3/7 inhibitor Ac-DEVD-CHO [43,49], for 30 min before exposure to 0.2 μM DOX or 0.2 μM MTX for 48 h. NR uptake and MTT reduction assays were performed after that exposure period.

### 4.4. Differentiated SH-SY5Y Cells

SH-SY5Y cells can be differentiated and a dopaminergic state is obtained after differentiation, while undifferentiated SH-SY5Y cell respond as catecholaminergic neurons [55]. For differentiating cells into a dopaminergic phenotype, cells (density 25,000 cells/cm^2^) were seeded in complete DMEM medium containing 10 nM RA for three days. At the third day, cells were then exposed to 80 nM TPA on complete DMEM medium and kept for another three days [48,49]. After the 6-day differentiation protocol, the cells were exposed to 0.2 μM DOX or MTX for 48 h, and the MTT reduction test was performed.

### 4.5. Cytotoxicity Evaluation

To compare DOX and MTX cytotoxicity and to determine if putative protectors could prevent against DOX or MTX-induced toxicity, two assays were used: the NR lysosomal uptake and the MTT reduction assays. Both methods were performed 24 or 48 h after the cells’ exposure to cytostatic drugs in 48-well plates. 

### 4.6. MTT Reduction Assay

The MTT colorimetric assay is based on the mitochondrial reduction of the tetrazolium salt and formazan formation. At the selected time-point, the cellular medium was changed and 200 μL of complete medium and 20 μL of MTT (5 mg/mL) were added to each well. A 3-h incubation period at 37 °C was then necessary to allow the reduction of MTT in both differentiated and undifferentiated SH-SY5Y cells. The medium was then removed and 200 μL of DMSO were added. The plate was shacked for 15 min until total dissolution of the formazans. Spectrophotometric measurement of the formazans formed was then done at 550 nm in a multi-well plate reader (Biotech Synergy HT, Winooski, VT, USA). The percentage of MTT reduction of control cells was set to 100% and the values of each treatment are expressed as percentage of control cells.

### 4.7. Neutral Red Lysosomal Uptake Assay

The NR uptake assay is based on the ability of viable cells to uptake the supravitally dye that penetrates cell membranes and concentrates in lysosomes. After a 48-h exposure time, the medium was removed and warm NR (33 μg/mL) enriched medium was placed in each well (250 μL/well). Plates were kept at 37 °C for 3 h, protected from light. The medium was then removed and the wells were washed with 250 μL of warm HBSS solution with calcium and magnesium. Thereafter, the HBSS solution was rejected and 200 μL of the lysis solution (50% ethanol/1% acetic acid) were added. The plate was shaken for 15 min, protected from light, until a homogeneous solution was obtained. The absorbance was read at two wavelengths, 540 and 690 nm (reference), on a multi-well plate reader (Biotech Synergy HT, Winooski, VT, USA) and results are presented as percentage of control cells, whose mean values were set to 100%.

### 4.8. Microscopic Evaluation of the Cells

#### 4.8.1. Phase Contrast Microscopy

In undifferentiated SH-SY5Y cells, phase-contrast microscopy morphological evaluation was performed in 12-well plates to determine the toxic effects of both cytostatic drugs after a 24- or 48-h exposure. An Nikon Eclipse TS100 inverted microscope equipped with a DS-Fi1 camera (Tokyo, Japan) was used. 

#### 4.8.2. Hoechst Staining

To evaluate the effects of MTX and DOX on the nuclear morphology of undifferentiated SH-SY5Y cells, the Hoechst staining was performed following a 24-h or a 48-h exposure to the drugs, as previously described [43]. Briefly, cells were fixed in 4% paraformaldehyde (10 min, 4 °C) and washed three times with PBS with calcium and magnesium. Cells were stained with the nuclear dye Hoechst 33258 (final concentration of 5 μg/mL) for 10 min at 37 °C (protected from light), and then washed three times, at room temperature, with PBS containing calcium and magnesium. Cells were examined in a Nikon Eclipse TS100 microscope equipped with a Nikon DS-Fi1 camera, using a standard fluorescein filter (λ_excitation_ = 346 nm and λ_emission_ = 460 nm) and then counted manually for total cells and condensed nucleuses.

#### 4.8.3. Ethidium Bromide and Acridine Orange Staining

The fluorescent DNA-intercalating dyes ethidium bromide and acridine orange are used to discriminate between necrotic and apoptotic cell death. Ethidium bromide intercalates with nucleic acids if the outer cellular membrane is ruptured. Acridine orange diffuses through intact membranes of live cells and largely accumulates in acidic vesicles. After a 48-h incubation with MTX or DOX, the medium was removed and the protocol was done as previously described [49]. Cells were examined in a Nikon Eclipse TS100 microscope equipped with a Nikon DS-Fi1 camera, using a standard fluorescein filter (λ_excitation_ = 485 nm and λ_emission_ = 525 nm).

#### 4.8.4. Evaluation of the Mitochondrial Membrane Potential

The evaluation of the mitochondrial membrane potential was also done as previously described [43]. Briefly, cells were incubated for 48 h with 0.13 μM MTX or 0.13 μM DOX and subsequently incubated for 30 min at 37 °C with DiO6 (35 nM/well). Each condition had a well without any DiO6 to evaluate whether any component of the medium or drugs tested emitted any residual fluorescence that could interfere with the readings. After the 30 min incubation time, cells were washed twice with warm PBS with calcium and magnesium and photographs were taken in a fluorescence microscope (Nikon Eclipse TS100 equipped with a Nikon DS-Fi1 camera), using a standard fluorescein filter (λ_excitation_ = 485 nm and λ_emission_ = 520 nm).

## 5. Statistical Analysis

The results are expressed as mean ± standard deviation. When the two molecules and several concentrations were compared at different concentrations, statistical analysis was performed using the two-way ANOVA test, followed by the Bonferroni *post-hoc* test, once a significant *p* value was reached. When dealing with three or more conditions, the D’Agostino & Pearson normality test was used to evaluate data distribution. A parametric analysis of variance (ANOVA) was performed when data distribution was normal, followed by the Tukey’s *post hoc* test. When data did not follow a normal distribution, statistical analysis was performed using the Kruskal-Wallis test, followed by the Dunn’s *post-hoc* test, once a significant *p* value was reached. Statistical significance was set at *p* < 0.05. All statistical analyses were performed using GraphPad Prism 7 software (GraphPad Software, La Jolla, CA, USA). All details of the statistical analyses can be found in the figure legends.

## Figures and Tables

**Table 1:** Number of cells and condensed nuclei after the Hoescht staining at 48 h.

Parameters				
**MTX**	**Control**	**0.13 μM**	**0.2 μM**	**0.5 μM**
**Condensed nuclei**	4 ± 4	205 ± 111	130 ± 29	117 ± 29
**Number of cells**	439 ± 102	357 ± 95	259 ± 19	212 ± 15
**Ratio of condensed nuclei/number of cells**	0.89 ± 0.76	57.04 ± 24.98 *	50.09 ± 9.30	54.87 ± 9.54
**DOX**	**Control**	**0.13 μM**	**0.2 μM**	**0.5 μM**
**Condensed nuclei**	2 ± 1	43 ± 8	26 ± 12	84 ± 14
**Number of cells**	436 ± 98	263 ± 29	186 ± 59	170 ± 18
**Ratio of condensed nuclei/number of cells**	0.39 ± 0.27	16.20 ± 2.05	13.53 ± 2.57	49.00 ± 4.00 **

Results are presented as mean ± SD of two independent experiments and two different fields each. Each field was counted manually and the microphotographs were taken with the magnification of 200×. Statistical analyses were performed on the ratio of condensed nuclei/number of cells using the Kruskal-Wallis test, followed by the Dunn’s *post-hoc* test. (* *p* < 0.05; ** *p* < 0.01 versus control).

## Abbreviations

BBB	Blood-brain barrier
CHX	Cycloheximide
CNS	Central nervous system
DMEM	Dulbecco’s modified Eagle medium
DOX	Doxorubicin
EMA	European Medicines Agency
HBSS	Hanks’ balanced salt solution
MS	Multiple sclerosis
MTT	3-(4,5-Dimethylthiazol-2-yl)-2,5-diphenyl tetrazolium bromide
MTX	Mitoxantrone
NAC	*N*-Acetyl-l-cysteine
NR	Neutral Red
PBS	Phosphate buffered saline
RA	Retinoic acid
TPA	12-*O*-tetradecanoylphorbol 13-acetate
